# A distinction between *Fritillaria Cirrhosa* Bulbus and *Fritillaria Pallidiflora* Bulbus via LC–MS/MS in conjunction with principal component analysis and hierarchical cluster analysis

**DOI:** 10.1038/s41598-023-29631-8

**Published:** 2023-02-15

**Authors:** Chuanlan Liu, Simei Liu, Wai Ming Tse, Kathy Wai Gaun Tse, Aga Erbu, Hai Xiong, Gongga Lanzi, Yanyong Liu, Bengui Ye

**Affiliations:** 1 Medical College of Tibet University, Lasa, 850002 People’s Republic of China; 2 Key Laboratory of Drug-Targeting and Drug Delivery System of the Education Ministry, Sichuan Engineering Laboratory for Plant-Sourced Drug, and Sichuan Research Center for Drug Precision Industrial Technology, West China School of Pharmacy, Sichuan University, Chengdu, 610041 People’s Republic of China; 3 Nin Jiom Medicine Manufactory (H.K.) Limited, 16/F. Block A, Texaco Road, Tsuen Wan, N.T., Hong Kong, People’s Republic of China

**Keywords:** Drug discovery, Plant sciences

## Abstract

*Fritillaria Cirrhosa* Bulbus (known as chuanbeimu in Chinese, FCB) is one of the most used Chinese medicines for lung disease. However, a variety of substitutes have entered the market, with *Fritillaria Pallidiflora* Bulbus (FPB) being the most common. Due to their similarity in appearance, morphology, and chemical composition but a large price difference, the FCB has frequently been adulterated with the FPB, posing a serious challenge to the distinction and quality of the FCB. Therefore, we aimed to distinguish FCB and FPB based on their main nine isosteroidal alkaloid contents and test the potential of chemometrics as a discrimination approach for evaluating quality. The nine major isosteroidal alkaloids were measured using a liquid chromatography with tandem mass spectrometry (LC–MS/MS) approach in 41 batches of FCB and 17 batches of FPB. Additionally, they were categorized and distinguished using the methods of hierarchical cluster analysis (HCA) and principal component analysis (PCA). Quantitative analysis revealed that the nine alkaloids were present in different amounts in the two types of Fritillariae bulbus. In FCB, the highest amount was peimisine (17.92–123.53 μg/g) and the lowest was delavine (0.42–29.18 μg/g), while in FPB, imperialine was higher (78.05–344.09 μg/g), but verticinone and verticine were less than the other seven alkaloids. The FCB and FPB were successfully classified and distinguished by the HCA and PCA. Taken together, the method has a good linear relationship (R^2^ > 0.9975). The LOD and LOQ of the nine alkaloids were in the range of 0.0651–0.6510 and 0.1953–1.9531 ng/mL, respectively. The intra- and inter-day precision were shown to be excellent, with relative standard deviations (RSDs) below 1.63% and 2.39%, respectively. The LC–MS/MS method in conjunction with HCA and PCA can effectively differentiate FCB and FPB. It may be a promising strategy for quality evaluation and control at the FCB.

## Introduction

According to the People's Republic of China Pharmacopoeia (ChP), there are six original plants of FCB, respectively: Fritillaria cirrhosa D. Don, Fritillaria unibracteata Hsiao et K.C. Hsia, Fritillaria przewalskii Maxim, Fritillaria delavayi Franch, Fritillaria taipaiensis P. Y. Li and Fritillaria unibracteata Hsiao et K.C. Hsia wabuensis (S.Y. Tang et S.C. Yue) Z.D. Liu, S. Wang et S.C. Chen. It is a precious Chinese herbal medicine that has been used for many years because of its pharmacological effects^1^ on clearing heat and moistening the lung^2^, cough suppressing^3^, eliminating phlegm^4,5^, and asthma calming^6^. Contemporary research has revealed that it has anti-inflammatory^7^, antioxidant^8,9^, analgesic^10^, antitumor^11^, anti-COVID-19^12^, and other properties. A growing amount of focus has been placed on it due to its outstanding medical value. However, market demand has outpaced supply, resulting in a price increase for FCB. On the herbal market, FCB has often been adulterated with FPB. Since they have a similar appearance, morphology, and chemical composition, a variety of substitutes have also appeared on the herbal market. It weakens the genuineness of Chinese medicinal materials and harms the curative effect of traditional Chinese medicine. Counterfeit plant products, especially those made by using incorrect plant materials in the pharmaceutical industry, have become a global problem. Therefore, a reasonable method is needed to identify the FCB for quality evaluation and control.

Phytochemical studies revealed that FCB contained isosteroidal alkaloids^13,14^, terpenoids, saponins^15^, nucleosides, volatile oils, organic acids, and sterols^16,17^. The composition and content reflect their pharmacological effects. Among them, alkaloids, as the main active ingredient, have been illustrated to exert significant therapeutic effects on many diseases. The content of isosteroidal alkaloids in cultivated and wild products, on the other hand, varies depending on the growing environment^18^ and plucking time^19–21^. The quality of traditional Chinese medicine is linked to its efficacy and safety. Thus, understanding the composition and content of isosteroidal alkaloids is crucial to distinguishing between FCB and its substitutes and ensuring their quality, thus playing a great role in promoting the development of traditional Chinese medicine.

In recent years, to distinguish between FCB and its substitutes, polymerase chain reaction (PCR)^22^, deoxyribose nucleic acid (DNA) barcoding^23–25^, and other biochemical techniques have been used. However, various fluorescence spectrum^26^ analytical techniques have also been used for the determination of isosteroidal alkaloids in Fritillariae bulbus, including thin-layer chromatography (TLC)^27^, ultraviolet (UV), near-infrared spectroscopy (NIR)^28,29^, gas chromatography (GC)^30^, high-performance liquid chromatography (HPLC) fingerprinting^31,32^, and ultra-high performance liquid chromatography quadrupole time-of-flight mass spectrometry (UPLC-ELSD)^33^. Among the methods mentioned above, TLC is not suitable for many assays. The main application of IR is qualitative analysis. The main application of GC takes a lot of time. The most popular technique is HPLC–UV. It is the most widely used method for phytochemical analysis, but it is not very effective because the isosteroidal alkaloids' skeleton lacks any useful chromophore. Low sensitivity characterizes the HPLC-ELSD method. Compared with the earlier-mentioned methods, LC–MS/MS has proved to be a powerful tool for the comprehensive analysis of multiple isosteroidal alkaloids in Fritillariae bulbus extracts.

Considering the above situation, there is not a highly efficient and sensitive method of differentiation to distinguish FCB and FPB. Meanwhile, no studies have been reported on the chemical differentiation between FCB and FPB via an LC–MS/MS method. Thus, the component differences in the development strategy and distinguishing the two kinds of Fritillariae bulbus need to be solved urgently. LC–MS/MS as an analytical technique for the detection and quantification of different analytes with highly specific, sensitive, selectivity, and rapid method, provides an efficient means for the measurement of analytes in biological matrices^34–36^. In LC–MS/MS analysis of such macromolecules, one charged form is selected as the precursor ion, which is then fragmented by collision-induced dissociation (CID) in MS/MS to generate product ions, a process referred to as "multiple-reaction monitoring” (MRM). The MRM method minimizes interference from endogenous molecules within biological matrices^37–40^. In this study, we compare the differentiation between FCB and FPB through the quantification of isosteroidal alkaloids content using LC–MS/MS and classify and identify these two types of Fritillariae bulbus using chemometric methods. It overcomes the shortcomings of UV, HPLC, and other methods and provides a more reliable method for the determination of active ingredients.

## Materials and methods

### Plant sample collection

For the research, a total of 41 batches of *Fritillaria cirrhosa* bulbus, 12 batches of *Fritillaria pallidiflora Schrenk* bulbus, and 5 batches of *Fritillaria walujewii Regel* bulbus were collected from wild or cultivated sources in various provinces and locations throughout China. According to the ChP, FPB includes *F. pallidiflora* or *F. walujwii,* namely, the bulbus of *Fritillaria pallidiflora Schrenk* and the bulbus of *Fritillaria walujewii Regel*. All the above samples were authenticated by Associate Professor Bengui Ye, and the voucher specimens were deposited in the herbarium of the West China School of Pharmacy at Sichuan University. The details of all samples, as well as their characteristics, are listed in Table 1.

**Table 1:** Sources and origins of two species of Fritillariae bulbus.

Sample code	Species	Source	Collecting time	Origin
Fc-c-1	*F. cirrhosa*	County Mao	15 September 2017	Cultivated
Fc-c-2	*F. cirrhosa*	Qinghai	30 July 2018	Cultivated
Fc-c-3	*F. cirrhosa*	Qinghai	30 July 2018	Cultivated
Fc-c-4	*F. cirrhosa*	Qinghai	22 January 2016	Cultivated
Fc-c-5	*F. cirrhosa*	Kangding	22 January 2016	Cultivated
Fc-c-6	*F. cirrhosa*	Qinghai	22 January 2016	Cultivated
Fc-c-7	*F. cirrhosa*	Kangding	22 January 2016	Cultivated
Fc-c-8	*F. cirrhosa*	County Mao	28 March 2016	Cultivated
Fc-c-9	*F. cirrhosa*	County Mao	01 November 2016	Cultivated
Fc-c-10	*F. cirrhosa*	County Mao	21 August 2018	Cultivated
Fc-c-11	*F. cirrhosa*	Qinghai	22 August 2017	Cultivated
Fc-c-12	*F. cirrhosa*	Tibet	August 2017	Cultivated
Fc-c-13	*F. cirrhosa*	Qinghai	20 March 2015	Cultivated
Fc-c-14	*F. cirrhosa*	Qinghai	25 October 2014	Cultivated
Fc-c-15	*F. cirrhosa*	County Mao	01.November 2016	Cultivated
Fc-c-16	*F. cirrhosa*	Qinghai	20 March 2015	Cultivated
Fc-c-17	*F. cirrhosa*	County Mao	28 March 2016	Cultivated
Fc-c-18	*F. cirrhosa*	County Mao	27 July 2016	Cultivated
Fc-c-19	*F. cirrhosa*	County Mao	27 July 2016	Cultivated
Fc-c-20	*F. cirrhosa*	County Mao	27 July 2016	Cultivated
Fc-c-21	*F. cirrhosa*	Qinghai	20 June 2018	Cultivated
Fc-c-22	*F. cirrhosa*	Qinghai	10 July 2018	Cultivated
Fc-c-23	*F. cirrhosa*	Qinghai	10 August 2018	Cultivated
Fc-c-24	*F. cirrhosa*	Qinghai	20 June 2018	Cultivated
Fc-c-25	*F. cirrhosa*	Qinghai	20 August 2018	Cultivated
Fc-c-26	*F. cirrhosa*	Qinghai	20 June 2018	Cultivated
Fc-c-27	*F. cirrhosa*	Qinghai	August 2017	Cultivated
Fc-w-1	*F. cirrhosa*	Songpan	04 January 2014	Wild
Fc-w-2	*F. cirrhosa*	Luhuo	29 June 2018	Wild
Fc-w-3	*F. cirrhosa*	Xiangcheng	July 2018	Wild
Fc-w-4	*F. cirrhosa*	Yajiang	01 July 2018	Wild
Fc-w-5	*F. cirrhosa*	Kangding	13 July 2015	Wild
Fc-w-6	*F. cirrhosa*	Kangding	June 2018	Wild
Fc-w-7	*F. cirrhosa*	Qinghai	21 August 2018	Wild
Fc-w-8	*F. cirrhosa*	Tibet	July 2017	Wild
Fc-w-9	*F. cirrhosa*	Changduzuogong	August 2018	Wild
Fc-w-10	*F. cirrhosa*	Ganzi	June 2018	Wild
Fc-w-11	*F. cirrhosa*	Yushu	June 2018	Wild
Fc-w-12	*F. cirrhosa*	Ganzi	July 2018	Wild
Fc-w-13	*F. cirrhosa*	Songpan	05 January 2019	Wild
Fc-w-14	*F. cirrhosa*	Qinghai	June 2016	Wild
Fp-c-1	*F. Pallidiflora*	Yili	June 2015	Cultivated
Fp-c-2	*F. Pallidiflora*	Yili	June 2015	Cultivated
Fp-c-3	*F. Pallidiflora*	Yili	15 May 2014	Cultivated
Fp-c-4	*F. Pallidiflora*	Yili	15 May 2014	Cultivated
Fp-c-5	*F. Pallidiflora*	Yili	06 October 2015	Cultivated
Fp-c-6	*F. Pallidiflora*	Yili	June 2015	Cultivated
Fp-c-7	*F. Pallidiflora*	Yili	June 2015	Cultivated
Fp-c-8	*F. Pallidiflora*	Yili	June 2015	Cultivated
Fp-c-9	*F. Pallidiflora*	Yili	June 2015	Cultivated
Fp-c-10	*F. Pallidiflora*	Yili	06 October 2015	Cultivated
Fp-c-11	*F. Pallidiflora*	Yili	15 May 2014	Cultivated
Fp-c-12	*F. Pallidiflora*	Yili	10 June 2016	Cultivated
Fw-c-1	*F. walujewii*	Tuoli	16 June 2016	Cultivated
Fw-c-2	*F. walujewii*	Tuoli	06 June 2016	Cultivated
Fw-c-3	*F. walujewii*	Tacheng	06 June 2016	Cultivated
Fw-c-4	*F. walujewii*	Xijiang	06 June 2016	Cultivated
Fw-c-5	*F. walujewii*	Tuoli	06 June 2016	Cultivated

### Main chemical reagents and apparatus

**Figure 1:**
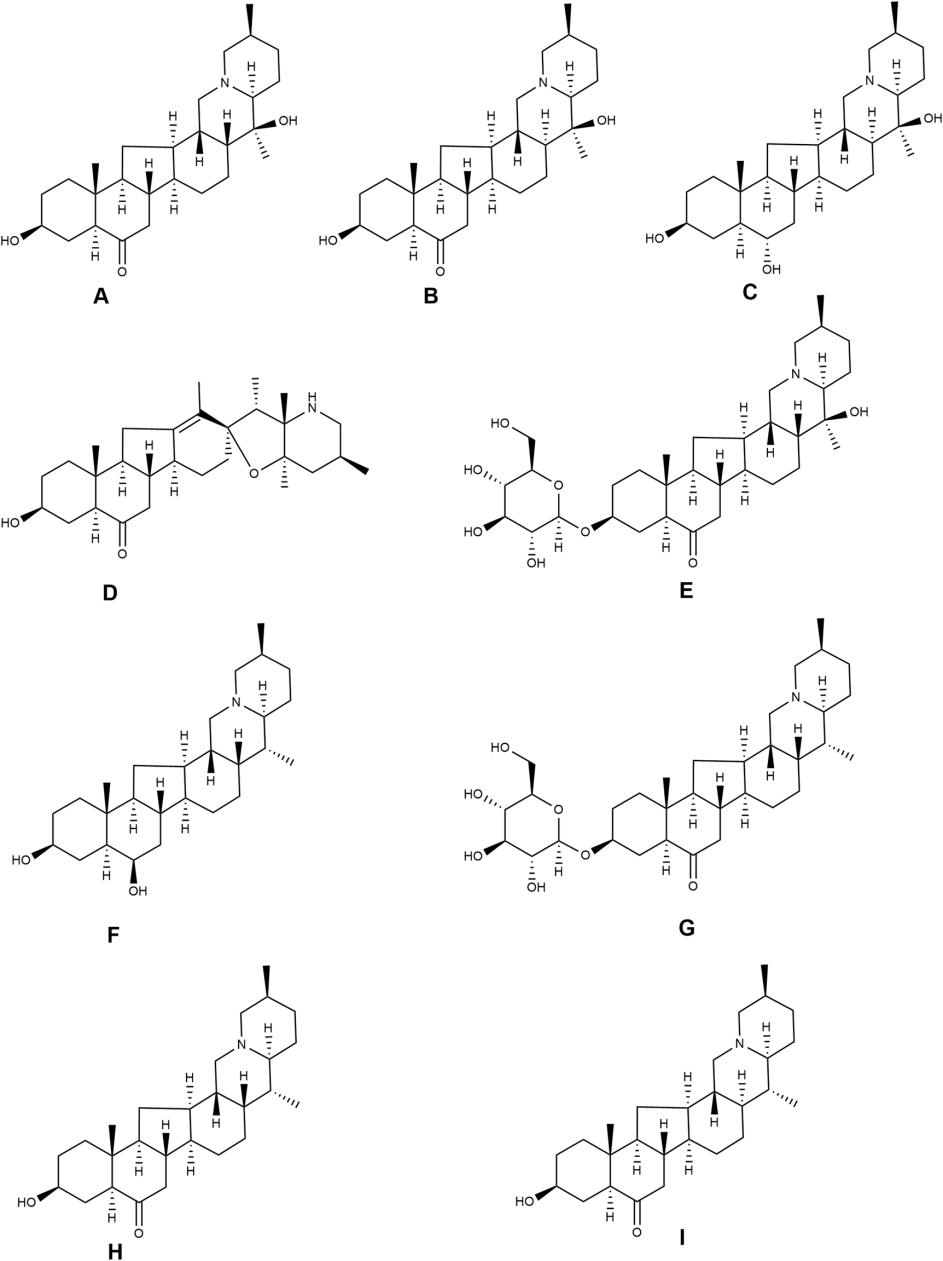
The chemical structure of nine alkaloids: (**A**) Imperialine; (**B**) Verticinone; (**C**) Verticine; (**D**) Peimisine; (**E**) Imperialine-3-β-d-glucoside; (**F**) Delavine; (**G**) Yibeinoside A; (**H**) Delavinone; (**I**) Ebeiedinone.

The standards of imperialine, verticinone, verticine, delavine, and peimisine were purchased from Chengdu Push Bio-Technology Co., Ltd. (Chengdu, Sichuan, China). Imperialine-3-β-d-glucoside was purchased from Chengdu Herbpurify Co., LTD. (Chengdu, Sichuan, China). Yibeinoside A, delavinone, and ebeidinone were purchased from Chengdu Must Bio-Technology Co, LTD (Chengdu, Sichuan, China). The purity of all these substances was above 98%: verticine (99.71%), verticinone (99.69%), peimisine (99.60%), ebeiedinone (99.63%), imperialine (99.93%), imperialine-3-β-d-glucoside (99.44%), delavine (98.57%), delavinone (99.16%), and yibeinoside A (98.71%). The chemical structures of these compounds are shown in Fig. 1. Sigma-Aldrich (St. Louis, MO, USA) supplied the HPLC-quality formic acid, LC–MS grade acetonitrile, and methanol. All the other reagents used in this experiment were of analytical grade and obtained from Chengdu Chron Chemicals Co., Ltd. (Chengdu, Sichuan, China).

Pure water was purified by a Milli-Q water purification system (Millipore, Bedford, MA, USA); the FA2004 electronic analytical balance (Shanghai Sunny Hengping Scientific Instrument Co., Ltd.); the Vortex-Genie 2 vortex oscillator (Scientific Industries, USA); the SCQ-200 ultrasonic cleaner (Shanghai Acoustic Spectrum Ultrasonic Equipment Factory); the LG-02 two-pack high-speed Chinese medicine grinder (Rui'an Baixin Pharmaceutical Equipment Factory); the DZKW-4 electronic thermostatic water bath (Beijing Zhongxing Weiye Instrument Co., Ltd.).

The Shimadzu Nexera UHPLC LC-30A Ultra Performance Liquid Chromatograph with On-line Degasser DGU-20A3, Binary High-Pressure Liquid Delivery Unit LC-30AD, Column Oven CTO-30A, and autosampler was used for the chromatography analysis (Shimadzu, Tokyo, Japan). The MS was carried out on an AB Sciex Triple Quad TM 5500 triple quadrupole mass spectrometer with an ESI source (AB Sciex, Foster City, CA, USA).

### Preparation of standard solutions

The following approach was used in the preparation of standard solutions: to begin, nine isosteroidal alkaloids were precisely weighed at 5.970 mg (imperialine), 4.940 mg (verticinone), 6.280 mg (verticine), 5.550 mg (imperialine-3-β-d-glucoside), 5.500 mg (delavine), 4.710 mg (peimisine), 4.930 mg (yibeinoside A), 5.140 mg (delavinone), and 5.170 mg (ebeidinone). The alkaloids were then ultrasonically dissolved in methanol in a 5 mL volumetric flask, and the volume was set to the mark. Finally, 2 mL of the original standard solution was absorbed, and methanol was added to a volume of 10 mL. All solutions were maintained at 4 °C in dark brown calibrated flasks before use.

### Sample preparation

The following measures were used to prepare the sample: the medicinal materials of bulbus were pulverized, filtered through with a No. 20 mesh sieve, and dried at 45 °C to a constant weight. The dry powder (0.3–0.5 g) was then steeped for 1 h in a 25% ammonia solution (1:1, v/v) before being extracted with 10 mL of chloroform–methanol (4:1, v/v) at 80 °C for 2 h under reflux. The extract was filtered and concentrated to dryness at 65 °C before being diluted in 2 mL of methanol. After dilution of the sample by 0.1 to 1 mL with a 50/50 methanol/water solution (v/v), it was centrifuged at 20,000 rpm for 10 min. The filtrate was used as the test solution after passing through a 0.22 μm microporous membrane before being injected into an LC–MS apparatus for analysis. If the concentration of the test solution exceeded the linear range, it was quantitatively diluted using a 50% methanol–water solution.

### Analytical methods

The LC–MS/MS analysis was performed in the following sequence concerning Yang's method^41^ and with appropriate adjustments.

Conditions for chromatography: With the column temperature at 40 °C, a Shim-pack XRODS column (2.2 m, 100 mm × 2.00 mm inner diameter) (Shimadzu) was used as a chromatographic column. The mobile phase consisted of 0.1% (v/v) formic acid (A) and acetonitrile (B) with a pH value of 2.66, and the gradient elution was performed as follows: 0.00–1.00 min, 0.0–15.0% B; 1.00–3.00 min, 15.0–25.0% B; 3.00–4.00 min, 25.0% B; 4.00–5.00 min, 25.0–95.0% B; 5.00–5.50 min, 95.0% B; 5.50–5.60 min, 95.0–15.0% B; 5.60–7.90 min, 15.0% B; 8.00 min, stop. The injection volume for analysis was 1 μL with a flow rate of 0.4 mL/min.

**Figure 2:**
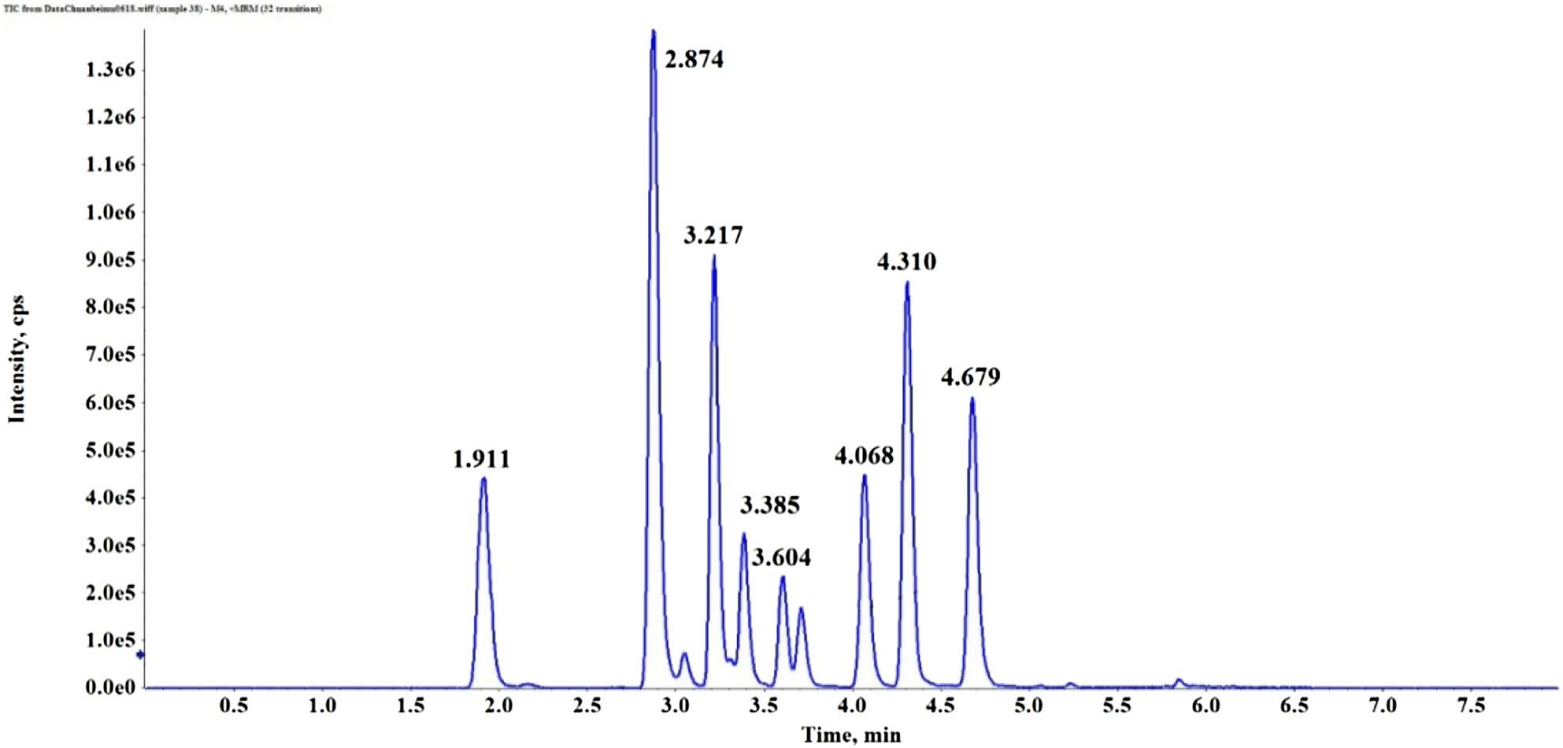
Total ions chromatogram of nine alkaloids in mixed standard solution using the multiple-reaction monitoring (MRM) mode.

The ESI–MS spectrum conditions were as follows: the injection voltage was set to 5.5 kV in the positive ion mode monitoring mode, and the ion source temperature was set to 500 °C. Nitrogen was used in all cases, atomized gas (Gas1) was used at 50.0 psi, heated gas (Gas2) at 50.0 psi, and curtain gas (CUR) at 40.0 psi. Multiple reaction monitoring (MRM) is used for scanning; the collision gas (CAD) pressure is 9.0, and the Q1 and Q3 resolutions are both UNIT. The MS detection parameters of nine isosteroidal alkaloids are shown in Table 2. All compounds were detected using multiple reaction monitoring (MRM) in positive mode. The total ion chromatograms (TICs) of nine isosteroidal alkaloids in a mixed standard solution are shown in Fig. 2. Data acquisition and processing were performed using Analyst Software 2.0, which was utilized for data collection and processing (AB Sciex).

**Table 2:** MS detection parameters of nine isosteroidal alkaloids.

Standard reference	Retention time (min)	Q1 mass (Da)	Q3 mass (Da)	Dwell time (msec)	DP (Volts)	CE (Volts)
Peimisine	3.217	428.3	114.1	20	230	40
Yibeinoside A	3.715	576.4	414.4	15	150	80
Ebeiedinone	4.679	414.4	91.1	15	130	110
Delavinone	4.310	414.4	98.1	15	40	65
Delavine	4.068	416.4	98.1	15	180	65
Imperialine-3-*β*-d-glucoside	1.911	592.4	574.4	15	40	75
Verticine	3.385	432.4	414.3	15	130	75
Verticinone	3.604	430.3	412.3	15	120	65
Imperialine	2.874	430.4	138.1	15	100	60

### Method validation

To make the mixed control solution work, add 50% methanol–water solution to each control, resulting in a concentration of 20 g/mL. Using the multiple dilution method, a series of concentration standard curve solutions were created from the mixed standard stock solution for calibration curves. The chromatographic peak areas were recorded by the previous conditions to construct a standard curve. Linearity was determined using correlation coefficients. After mixing equal volumes of the sample solutions, the mixed sample solution was obtained. The mixed sample solution was injected into the LC–MS/MS system every 5 min to compensate for ionization and matrix effect fluctuations. The average content was calculated after testing each sample three times. Using the corresponding calibration curves, the concentrations of nine alkaloids were quantified. The findings were reported in micrograms of analyte per gram of herb (μg/g). All calibration curves were constructed from the peak area ratio of the tested reference peak to the internal standard vs their concentrations. The limit of detection (LOD) and limit of quantification (LOQ) for each analyte were defined by the concentration that generated peaks with signal-to-noise values of 3 and 10, respectively.

The precision of the developed method was determined by the intra- and inter-day variations. For the intra-day test, the samples were analyzed six times on the same day, while for the inter-day test, the samples were examined in duplicate on three consecutive days. The relative standard deviation (RSD) was calculated as a measure of precision. The chromatographic peak areas of nine alkaloids were measured, and the content and RSD values were calculated for each alkaloid.

To confirm the repeatability, six replicates of the same samples (FC-c-11) were extracted and analyzed. The chromatographic peak areas of nine alkaloids were measured, and the content and RSD values were calculated for each alkaloid.

A recovery experiment was performed to evaluate the accuracy. Each of the 9 parts of FC-c-11 was accurately weighed at about 0.5 g and divided into 3 parts. The mixed reference solution with high, medium, and low levels was added so that the amount of the final reference was equivalent to 80%, 100%, and 120% of the original amount of each component in the tested medicinal material. The average recoveries and RSD values were calculated at high, middle, and low concentrations, respectively. Each analyte's recovery was calculated as follows: recovery (%) = (detection amount − original amount)/amount added × 100.

The same sample solutions (FC-c-11) were tested for stability at room temperature for 0, 12, 24, 36, 48, and 72 h, and the RSD values for each time point were calculated.

### Statistical analysis

The results of the heatmap and hierarchical cluster analysis (HCA) were carried out using Multi Experiment Viewer 4.9.0 software (https://sourceforge.net/projects/mev-tm4/files/mev-tm4/). Principal component analysis (PCA) was performed using Simca 13.0 (Umetrics, Umea, Sweden). When the concentrations of investigated compounds were below the quantitation limit, the values of such elements were 0. All analyses were used to interpret sample differences based on the nine alkaloid contents.

### Ethics declarations

This article does not contain any studies involving human participants or animals performed by any of the authors.

### Informed consent

Informed consent was obtained from all individual participants included in the study.

## Results

### Method validation

The ultra-performance liquid chromatography-electrospray ionization mass spectrometry (UPLC-ESI–MS) method was validated under European Medicines Agency (EMEA) guidelines for analytical method validation (Quality Guidelines: Validation of Analytical Procedures Text and Methodology [ICH Q2]). The calibration curves exhibited good linearity (R^2^ > 0.9975) within the test range. For the nine alkaloids, the LOD and LOQ were 0.0651 ~ 0.6510 ng/mL and 0.1953 ~ 1.9531 ng/mL, respectively. The intra-day and inter-day precisions were both excellent, with RSDs of less than 1.63% and 2.39%, respectively. The repeatability presented as RSD was in the range of 0.65% to 1.99%. The recovery of all analytes varied between 96.91 and 101.41% with an RSD < 1.99%. The RSD values of the peak areas of the nine alkaloids in the 0, 12, 24, 36, 48, and 72 h determinations were less than 2.20%.

### The determination of nine isosteroidal alkaloids

**Figure 3:**
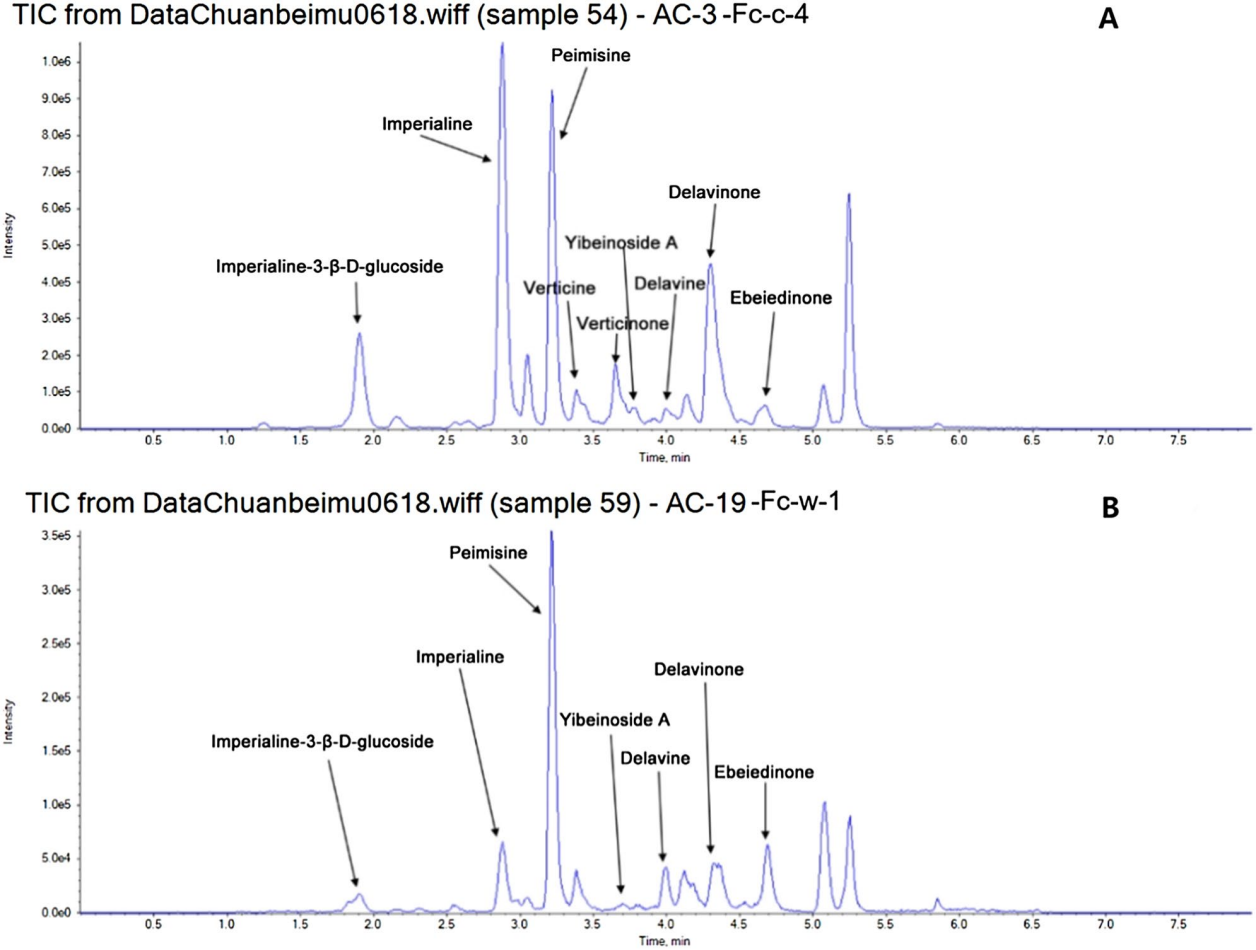
Representative total ions chromatograms of cultivated bulbus (**A**) and wild bulbus (**B**) of *Fritillaria cirrhosa* D. Don.

**Figure 4:**
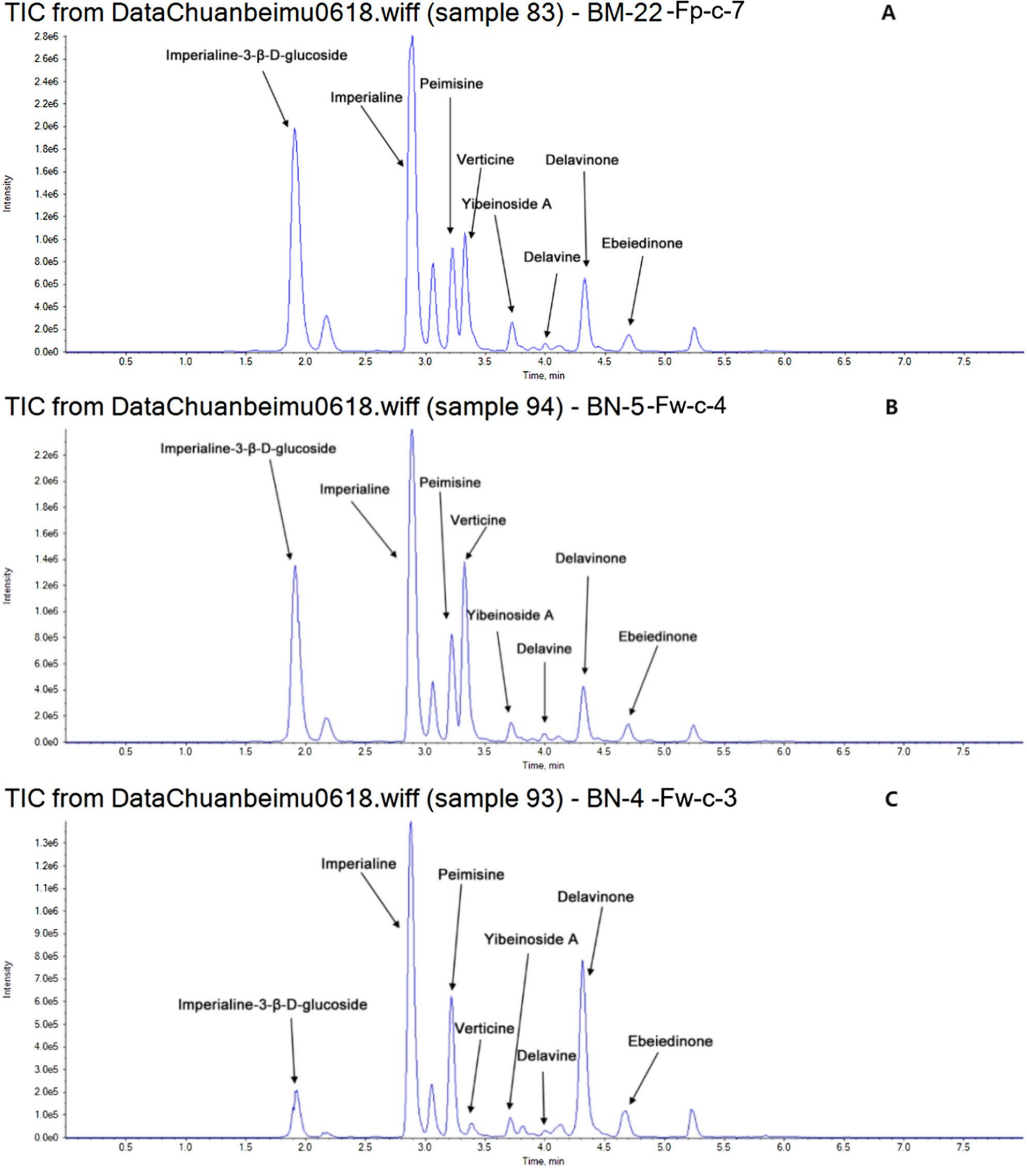
Representative total ions chromatograms of bulbus *Fritillaria pallidiflora Schrenk*.

This study measured the levels of nine isosteroidal alkaloids in FCB and FPB, including imperialine, verticine, verticinone, peimisine, imperialine-3-D-glucoside, yibeinoside A, delavinone, and ebeiedinone. The quantization for each sample is shown in Table 3. Figure 3 shows the representative total ion chromatograms of cultivated bulbus (A) and wild bulbus (B) from FCB. Figure 4 depicts the representative total ion chromatograms of FPB.

**Table 3:** The content of nine alkaloids in Fritillariae bulbus.

Sample name	Peimisine	Yibeinoside A	Ebeiedinone	Delavinone	Delavine	Imperialine 3-β-D-glucoside	Verticine	Verticinone	Imperialine
Fc_c_1	83.06	7.59	7.48	29.40	1.51	16.52	6.32	4.13	33.58
Fc_c_2	81.35	20.64	0.31	71.79	2.52	36.58	1.31	21.36	81.93
Fc_c_3	123.53	28.54	10.50	68.94	4.71	68.71	1.69	22.11	72.41
Fc_c_4	55.08	11.76	3.11	60.87	14.37	35.65	1.57	17.26	1.09
Fc_c_5	113.48	14.09	3.34	43.41	1.25	5.97	3.92	3.43	17.42
Fc_c_6	76.35	10.09	6.73	58.85	7.57	19.40	1.47	16.67	50.16
Fc_c_7	55.18	7.82	7.45	57.23	2.97	11.82	7.23	5.43	29.97
Fc_c_8	74.51	5.68	0.43	42.71	29.18	5.02	6.11	19.58	43.92
Fc_c_9	67.03	10.26	16.85	64.12	19.05	27.96	13.06	23.46	56.83
Fc_c_10	34.53	12.44	18.88	3.09	4.78	29.69	35.46	35.38	77.07
Fc_c_11	25.95	36.16	31.62	46.87	6.09	37.45	59.32	71.36	37.97
Fc_c_12	52.26	5.41	13.52	54.18	4.88	3.55	1.21	3.24	23.07
Fc_c_13	33.05	4.78	16.38	41.89	13.04	1.74	1.50	4.69	21.10
Fc_c_14	24.75	9.13	5.04	9.76	1.37	2.62	5.22	3.65	4.74
Fc_c_15	71.26	3.70	22.36	48.30	0.87	3.78	18.93	23.38	23.37
Fc_c_16	17.92	3.61	2.38	7.28	0.84	1.54	2.94	7.17	8.85
Fc_c_17	28.96	4.14	0.30	29.83	0.76	2.67	1.19	12.61	39.26
Fc_c_18	23.30	15.76	58.90	44.54	5.38	15.64	45.11	81.77	8.31
Fc_c_19	23.62	6.74	10.91	11.63	6.32	8.05	54.03	34.15	6.59
Fc_c_20	69.78	21.99	55.30	26.63	8.63	17.64	46.07	78.13	16.65
Fc_c_21	98.01	13.26	11.59	47.97	1.25	18.22	0.00	12.26	40.62
Fc_c_22	113.01	17.51	14.82	72.29	2.52	25.83	0.00	17.65	65.75
Fc_c_23	108.89	12.07	10.39	66.32	2.58	30.93	0.00	21.55	96.87
Fc_c_24	97.67	23.77	6.30	44.05	2.15	55.20	1.34	14.47	57.11
Fc_c_25	91.74	22.78	18.44	64.61	2.52	31.13	46.30	41.85	54.65
Fc_c_26	103.93	14.22	15.04	57.69	5.59	16.76	22.10	22.79	64.24
Fc_c_27	62.01	3.45	4.47	20.27	3.38	7.56	1.11	4.18	37.68
Fc_w_1	47.04	5.45	0.32	6.51	1.03	3.34	0.00	0.90	5.18
Fc_w_2	30.71	3.88	0.43	0.91	0.58	0.00	0.00	2.17	1.24
Fc_w_3	116.30	7.80	10.91	50.66	0.67	0.00	5.52	6.44	5.74
Fc_w_4	49.21	4.80	7.57	37.29	5.52	1.97	2.06	9.43	17.37
Fc_w_5	37.66	2.95	0.68	3.50	0.74	0.00	3.73	2.60	1.59
Fc_w_6	91.88	4.07	19.58	16.90	1.42	2.42	2.99	1.28	2.80
Fc_w_7	47.00	9.14	7.02	44.16	1.81	0.57	5.28	6.17	21.94
Fc_w_8	42.90	7.89	10.21	58.18	0.83	7.03	21.35	23.16	29.27
Fc_w_9	67.85	5.54	12.35	38.70	0.65	1.17	36.33	36.03	10.16
Fc_w_10	90.15	5.30	15.02	56.17	2.21	0.00	2.70	77.87	14.35
Fc_w_11	30.49	3.78	4.60	14.08	1.39	0.00	1.62	2.54	1.57
Fc_w_12	41.48	3.15	10.86	8.89	0.87	0.00	0.00	0.00	1.42
Fc_w_13	31.36	3.67	11.12	4.23	0.74	0.00	2.45	1.00	3.22
Fc_w_14	71.35	0.52	14.11	0.65	0.42	0.89	0.00	0.00	1.28
Fp_c_1	105.35	17.78	54.98	123.44	3.44	61.90	1.57	2.10	246.89
Fp_c_2	49.58	116.49	77.97	158.10	8.25	350.21	1.54	1.98	340.43
Fp_c_3	48.16	38.03	39.77	114.81	5.09	129.20	1.74	1.26	309.26
Fp_c_4	63.37	81.26	82.51	153.40	6.58	288.45	1.35	2.35	332.43
Fp_c_5	28.73	84.43	48.27	50.29	3.34	413.30	1.56	1.37	184.13
Fp_c_6	106.82	382.00	61.94	124.20	7.35	1180.07	2.02	1.83	312.01
Fp_c_7	88.39	209.41	36.46	107.81	7.59	823.50	2.40	1.55	316.98
Fp_c_8	77.37	98.37	86.68	148.37	7.47	316.20	12.86	7.61	344.09
Fp_c_9	62.69	86.72	52.73	127.42	6.51	323.61	1.59	1.78	293.44
Fp_c_10	26.09	63.68	43.24	57.09	2.89	259.65	1.97	1.61	183.82
Fp_c_11	37.63	63.10	33.14	71.93	5.17	332.64	1.38	1.48	247.69
Fp_c_12	54.72	46.15	29.52	92.76	4.40	1.38	1.83	41.18	243.97
Fw_c_1	65.90	82.62	68.55	82.76	1.94	577.56	1.25	1.32	248.61
Fw_c_2	216.05	144.76	55.89	279.53	27.11	61.69	0.00	0.00	78.05
Fw_c_3	100.98	55.42	27.77	121.90	6.82	1.02	1.92	0.00	111.40
Fw_c_4	58.89	112.31	30.71	71.09	3.72	1.51	1.65	1.29	226.88
Fw_c_5	234.59	95.20	41.21	231.47	27.35	73.56	1.32	1.23	149.66

Among the 41 batches of FCB, the highest level of peimisine (17.92–123.53 μg/g) was found overall, followed by delavinone (0.65–72.29 μg/g) and imperialine (1.09–96.87 μg/g). The lowest content was found in delavine (0.42–29.18 μg/g) and verticine (0.00–59.32 μg/g). The levels of ebeiedinone and yibeinoside A were also generally low, but both were detected. The levels of imperialine-3-β-d-glucoside and verticinone fluctuated significantly. In wild bulbus, the levels of yibeinoside A, delavine, imperialine-3-β-d-glucoside, verticine, verticinone, and imperialine were generally lower than those of the cultivated product. However, seven batches did not contain imperialine-3-β-d-glucoside, four batches did not contain verticine, and two batches did not contain verticinone, and the concentrations of delavine and verticine in the wild bulbus were typically less than 5 μg/g. All nine alkaloids were detected except for three batches of cultivated bulbus in which no verticine could be detected.

**Figure 5:**
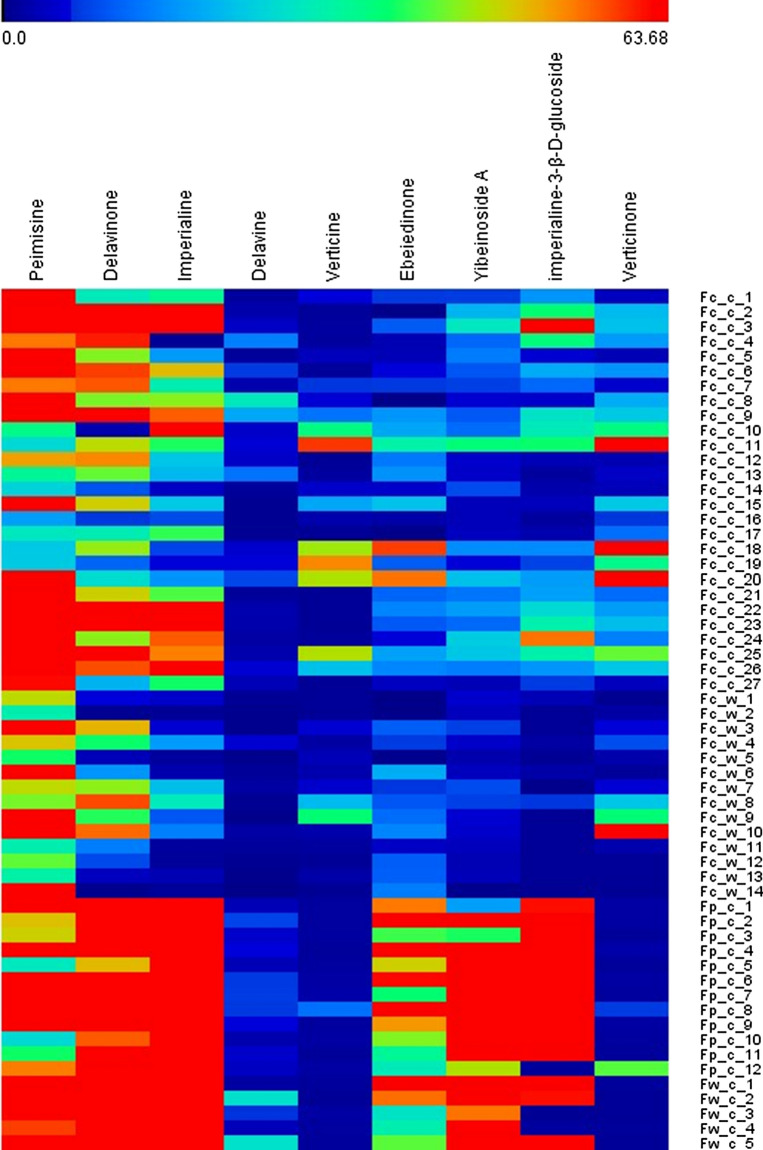
The heatmap shows the content of nine isosteroidal alkaloids in the bulbus of *F. cirrhosa*, *F. pallidiflora*, and *F. walujewii*. The transition from blue to red indicates an increasing amount of isosteroidal alkaloids.

The contents of verticinone and verticine in the 17 batches of FPB were less than those of the other seven alkaloids. All samples contained verticine and verticinone except for the Fw-c-3 samples, which did not contain verticinone, while the Fw-c-4 samples did not contain both verticine and verticinone. The next lowest component was delavine, with values ranging from 1.94–6.82 μg/g. Except for Fp-c-12, Fw-c-3, and Fw-c-4, which had low levels of imperialine-3-β-d-glucoside, all samples contained high levels of imperialine, imperialine-3-β-d-glucoside, delavinone, yibeinoside A, and peimisine, particularly imperialine, imperialine-3-β-d-glucoside, and delavinone, with concentrations ranging (111.40–344.09 μg/g), (129.20–577.56 μg/g), and (71.09–158.10 μg/g). Imperialine-3-β-d-glucoside of Fp-c-6 with up to 1180.07 μg/g. With a small fluctuation range and a relatively average content, ebeiedinone's content was second only to the above five alkaloids. The content of the nine isosteroidal alkaloids is intuitively shown by a heatmap in Fig. 5.

### Hierarchical cluster analysis

**Figure 6:**
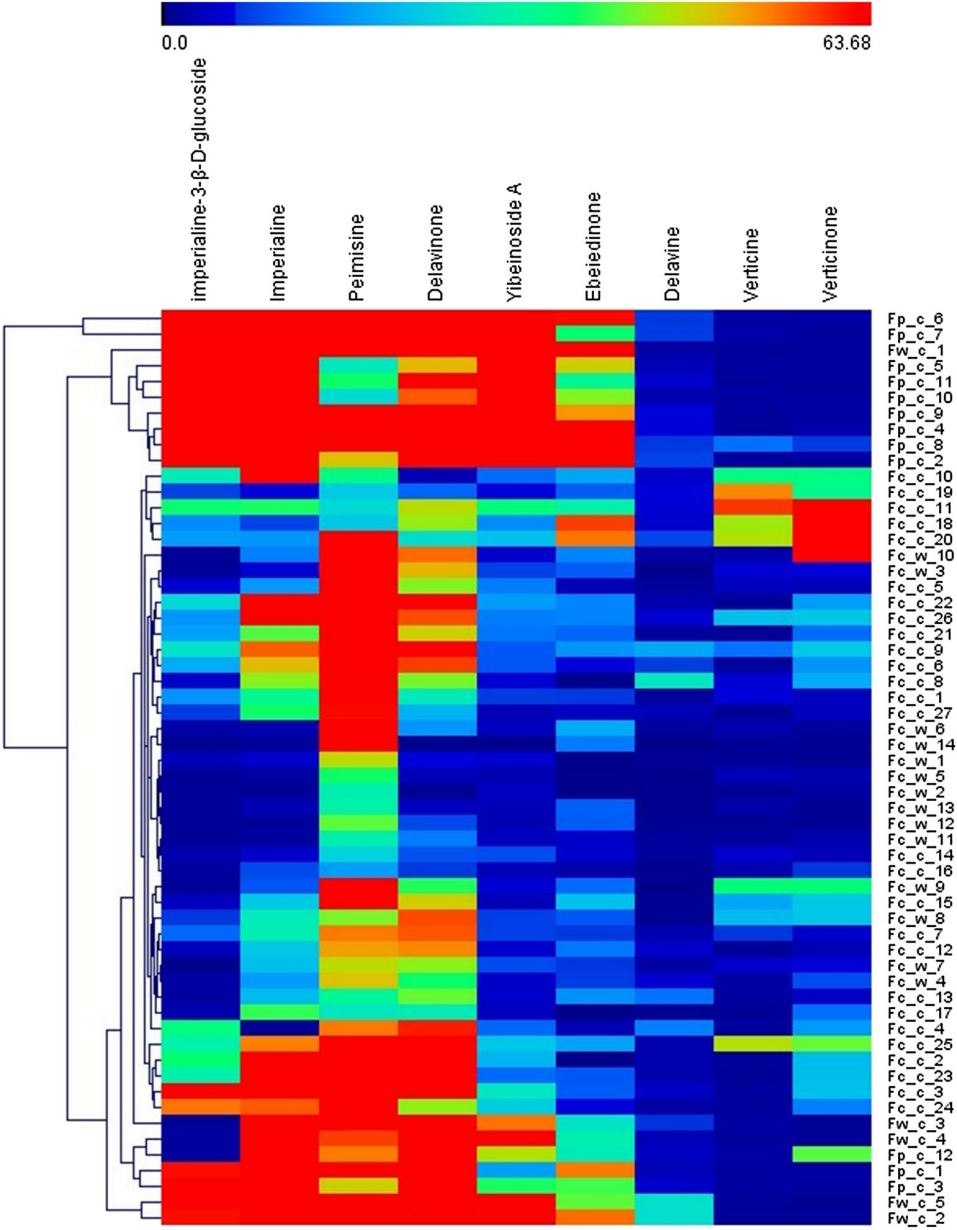
The results of a hierarchical cluster analysis on each sample are based on the high and low levels of each alkaloid.

HCA is a process that groups and categorizes large datasets based on qualitative or quantitative characteristics of experimental data to understand the internal relationships of datasets. Hierarchical cluster analysis was performed according to the content of each alkaloid in each sample, and the results are shown in Fig. 6. Of the 58 batches of samples, the first level of clustering is Fc-c-6 and Fc-c-7. In the second level, Fw-c-1, Fp-c-5, Fp-c-11, Fp-c-10, Fp-c-9, Fp-c-4, Fp-c-8, and Fp-c-2 were clustered into one group. As shown in Fig. 6, on the third hierarchical clustering, most of the FCB samples were grouped, distinguishing them from the FPB samples. From the HCA heatmap, we observed that for FCB, it was not easy to differentiate cultivated samples from wild samples.

### Reporting PCA results

**Figure 7:**
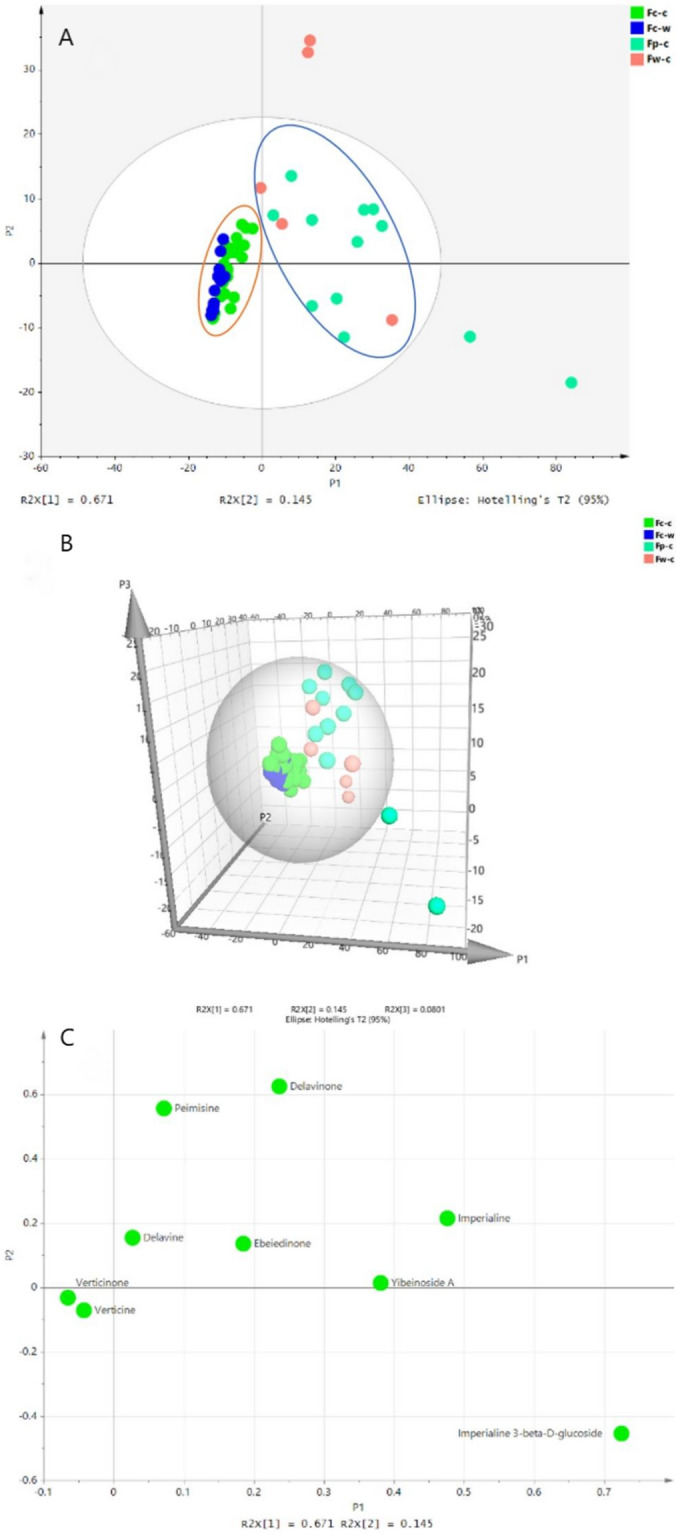
The PCA of the bulbus of *F. cirrhosa* and *F. pallidiflora* based on the content of nine alkaloids. Two-dimensional score plot (**A**) and loading plot (**C**), three-dimensional score plot (**B**).

PCA is a common unsupervised multivariate statistical analysis mainly used to examine differences between and within groups of samples. This method aims to reduce the dimensionality of the original data to visualize the clustering, trend, and outliers among the observations. In this study, we used the statistical analysis method of principal component analysis (PCA) to differentiate and cluster the FCB and FPB based on their isosteroidal alkaloid contents. The results are shown in Fig. 7.

As shown in Fig. 7A and B, the three principal components (PC1, PC2, and PC3) could account for 89.61% of the total variance. PC1 accounted for 67.1% of the variance, PC2 accounted for 14.5% of the variance, and PC3 accounted for 8.01% of the variance. Except for a few abnormal discrete samples, they can be roughly clustered into two domains: samples of FCB were grouped in the orange circle, while samples of FPB were grouped in the blue circle. The cultivated and wild FCB were placed close together in the orange circle, indicating that their isosteroidal alkaloid content was very similar. In Fig. 7C, PC1 was highly correlated with imperialine-3-β-D-glucoside, followed by imperialine and yibeinoside A, which were positively correlated with PC1, whereas verticinone and verticine were small and negatively correlated. PC2 was positively correlated with delavinone, whereas yibeinoside A and verticinone were found to be small and negatively correlated, implying that the two kinds of FCB could be distinguished from each other based on the content of nine isosteroidal alkaloids.

## Discussion

In this study, we successfully established an LC–MS/MS method for the simultaneous determination of nine common alkaloids in fritillaria. The method has a good linear relationship, high sensitivity, good precision, good repeatability, good stability, and high accuracy. It provides a rapid, convenient, and accurate method for the determination of alkaloids in fritillaria. Zhang and Hua^42,43^ can refer to this to make up for the lack of their research.

The results of HCA showed that FCB could be clustered into one group alone but could not distinguish between cultivated and wild samples. The variation in alkaloid content between FPB is wide, making it difficult to categorize.

To make up for the lack of HCA, we used PCA to explore the similarities and differences between the two species. The score plot in PCA identifies the distribution and clustering of samples, and the loading plot identifies the contribution of each variable to each principal component, which is the key to the clustering of samples in the score plot. In this study, each variable represents the content of nine alkaloids. According to the results of the content determination of nine alkaloids, the PCA method can effectively distinguish FCB from its substitute (FPB) and successfully solve the practical problem that it is difficult to identify them. In addition, according to their respective load diagrams, we can see the effects of nine alkaloids on the classification of different fritillaria and provide a scientific reference for the identification of fritillaria varieties (Supplementary Information).

The content of methods based on triple quadrupole tandem mass spectrometry has been widely used and reported as highly selective and sensitive methods for quantifying substances in herbal medicines^44^. However, it was limited to targeted components due to the difficulties of optimizing the multiple reaction monitoring transitions without authentic standards^45^. Naturally, those methods frequently necessitate longer analysis times, large samples must be analyzed, and various chemometric models^46^ must be used to validate and improve the current research findings. Compared with the method of the previous study^47^, the sample size and data in this paper are, however, objectively constrained. It is necessary to collect more samples according to different ecotypes and varieties to obtain more and more comprehensive detection data utilization^48^. In this way, a more accurate and reliable identification model for FCB was constructed.

## Conclusions

In the present study, we successfully established an LC–MS/MS method coupled with chemometric analysis for distinguishing FCB and FPB based on the nine isosteroidal alkaloids (imperialine, verticine, verticinone, peimisine, imperialine-3-β-d-glucoside, delvine, yibeinoside A, delavinone, and ebeiedinone) with good linearity, sensitivity, precision, reproducibility, stability, and accuracy. In summary, we used LC–MS/MS to perform a rapid and simultaneous quantitative analysis of nine isosteroidal alkaloids in 8 min, and it successfully provided scientific support for the quality control of FCB. Furthermore, this method is also likely to generate new ideas for quantitative analysis and identification of Fritillariae Bulbus.

## Supplementary Information

Supplementary Information.

## Data Availability

The datasets supporting the conclusions are included within this article or are available from the corresponding author upon request. We have permission to collect the plant samples under study. The study methods comply with Chinese guidelines.
