# Supplementary material for: A distinction between Fritillaria Cirrhosa Bulbus and Fritillaria Pallidiflora Bulbus via LC–MS/MS in conjunction with principal component analysis and hierarchical cluster analysis
Source: Sci Rep. 2023 Feb 15;13:2735. doi: 10.1038/s41598-023-29631-8 (PMC9931167; doi:10.1038/s41598-023-29631-8)
Supplement: Supplementary file 1 — Supplementary Information. [file 41598_2023_29631_MOESM1_ESM.doc]

**Supplementary material**

**Full Title:** **A distinction between** ***Fritillaria Cirrhosa* Bulbus and *Fritillaria Pallidiflora* Bulbus via LC-MS/MS in conjunction with principal component analysis and hierarchical cluster analysis**

Chuanlan Liu ^1, 2^, Simei Liu ^2^, Wai Ming Tse ^3^, Kathy Wai Gaun Tse ^3^, Er-bu AGA ^1, 2^, Hai Xiong ^1^, Ga Lan Zi Gong ^1^, [Yanyong Liu](https://pubmed.ncbi.nlm.nih.gov/?term=Liu+Y&cauthor_id=32804097) ^1^, and Bengui Ye * ^1, 2^.

^1^ Medical College of Tibet University, Lasa, 850002, P. R. China

^2^ Key Laboratory of Drug-Targeting and Drug Delivery System of the Education Ministry and Sichuan Province, Sichuan Engineering Laboratory for Plant-Sourced Drug and Sichuan Research Center for Drug Precision Industrial Technology, West China School of Pharmacy, Sichuan University, Chengdu, 610041, P.R. China

^3^ Nin Jiom Medicine Manufactory (H.K.) Limited,16/F, Block A, Texaco Road, Tsuen Wan, N.T., Hong Kong, P.R. China

**The first author:** Chuanlan Liu, The Medical College of Tibet University, Lasa, 850002, P. R. China. Email: lclyaoxuesc@163.com

**Correspondence:** Bengui Ye*, Key Laboratory of Drug-Targeting and Drug Delivery System of the Education Ministry and Sichuan Province, Sichuan Engineering Laboratory for Plant-Sourced Drug and Sichuan Research Center for Drug Precision Industrial Technology, West China School of Pharmacy, Sichuan University, No.17, Section 3, Renmin Road South, Chengdu 610041, China. Tel/Fax: 86-28-8550 3950. Email: [benguiye513@163.com](mailto:benguiye513@163.com)

**Bengui Ye will handle correspondence at all stages of refereeing and publication, as well as post-publication.**

**Funding information:**

This work was funded by the Major science and technology research project in 2021 from Tibet Science and Technology Department; Research on the product transformation of Tibetan genuine medicinal material (Tibetan Fritillaria Bulb) in the treatment of the chronic obstructive pulmonary disease (COPD) (Project number: XZ202101ZD0021G); the Special fund for strategic cooperation between Sichuan University and Dazhou Municipal Government (Project number: 2021CDDZ-13); the Sichuan University--Jiangsu Hengrui Pharmaceuticals West China Personnel Training and Discipline Development Funding (Project number: 2018029); the Science and Technology Major Project of Tibetan Autonomous Region of China (Project number: XZ202201ZD0001G); the National Natural Science Foundation of China; project name: The mechanism of hypoxic induced peripheral nerveremyelination in sound acclimatization under high altitude hypoxia environment (Project number: 81860567); the National Natural Science Foundation of China; project name: Study on the mechanism of central NFAT5 abnormality mediating HIBD epilepsy after high altitude hypoxia (Project number: 82060588) and the central government supports the talent development plan for local Everest scholars - the development plan for young doctors - the high-level talent cultivation project of Tibet University; Project name: Study on the effect of high altitude hypoxia on cochlear function (Project number: zdbs202216).

**Supplementary Table**

**Table 1.** Linear regression data, linear range of nine isosteroidal alkaloids

| compounds | Regression equation | R^2^ |
| --- | --- | --- |
| Peimisine | Y=583277X-4637.2 | 0.9994 |
| Yibeinoside A | Y=123173X-5993.2 | 0.9989 |
| Ebeiedinone | Y=357471X-381.23 | 0.9997 |
| Delavinone | Y=452719X-1580.7 | 0.9997 |
| Delavine | Y=491076X-4806.3 | 0.9991 |
| Imperialine-3-*β*-D-glucoside | Y=36376X+45.303 | 0.9993 |
| Verticine | Y=80297X-1351.1 | 0.9975 |
| Verticinone | Y=76389X-933.17 | 0.9991 |
| Imperialine | Y=351515X-5675.2 | 0.9992 |

**Supplementary Figure**


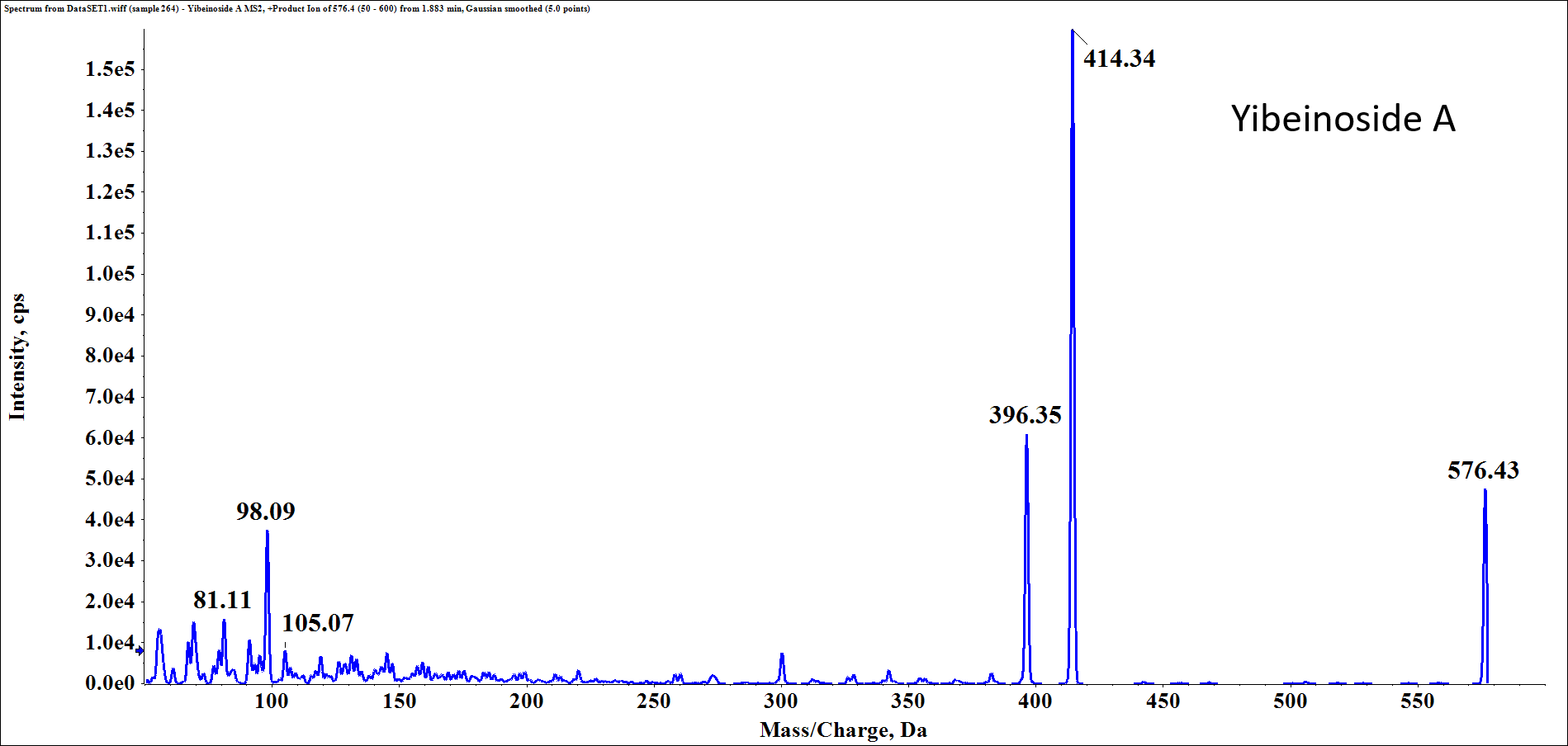


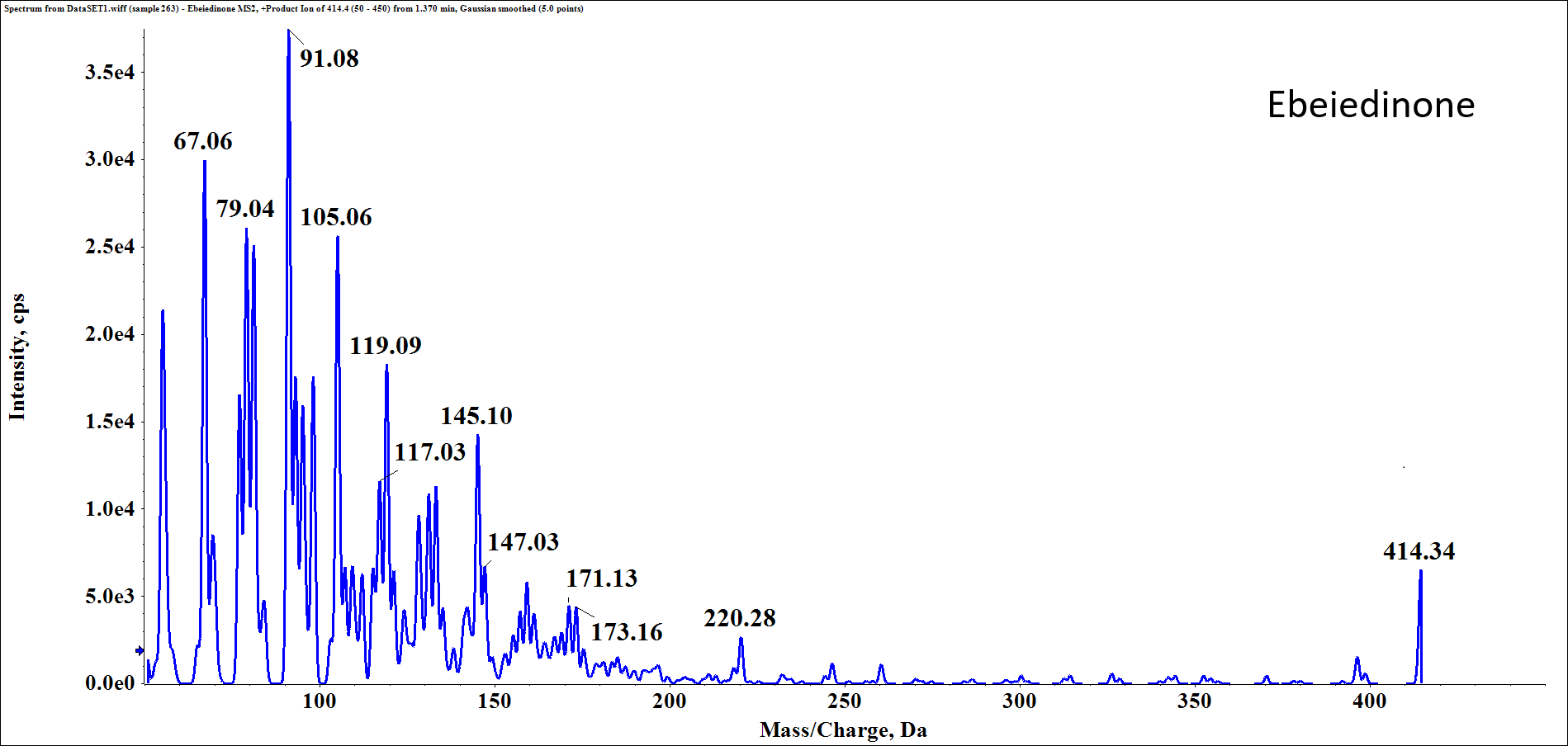


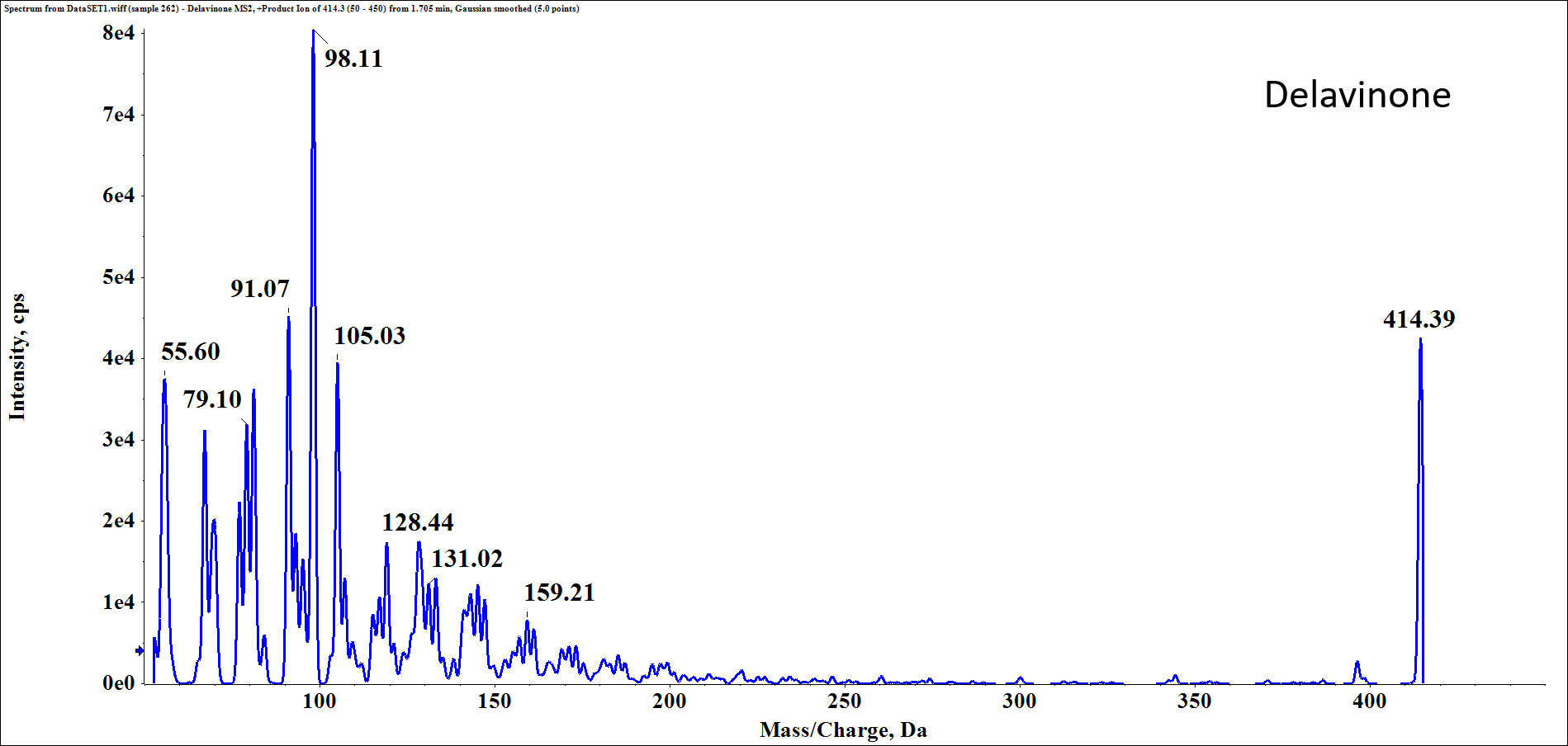


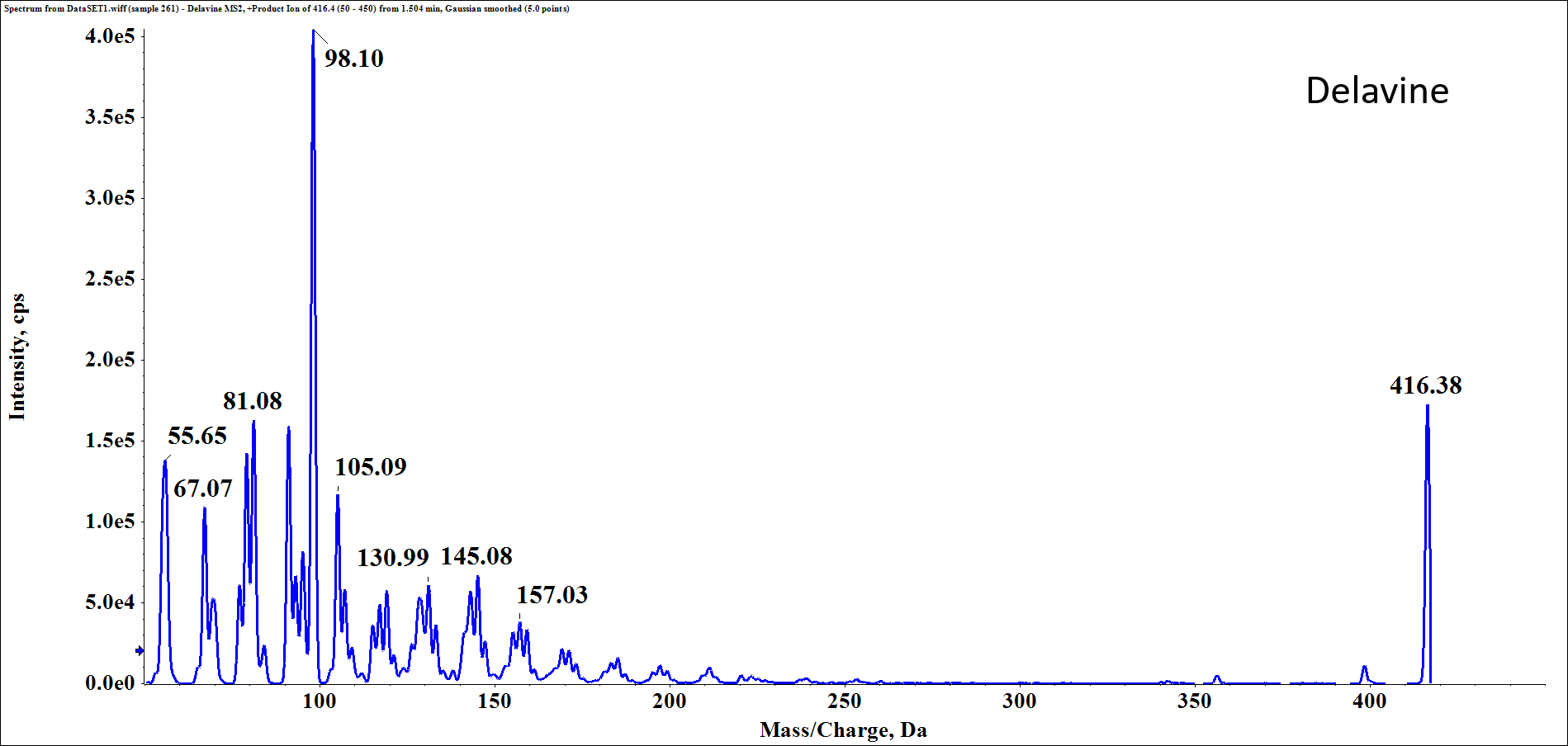


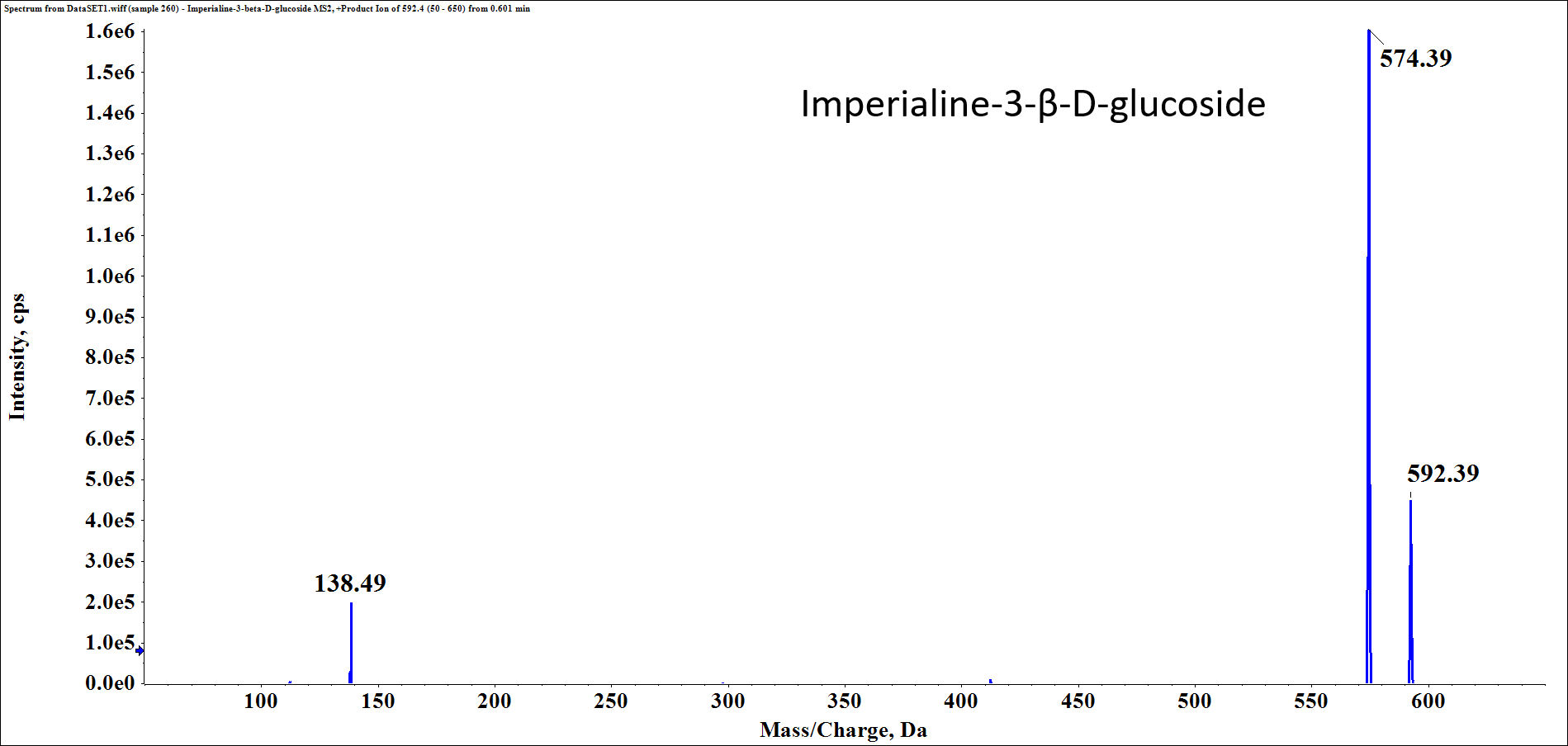


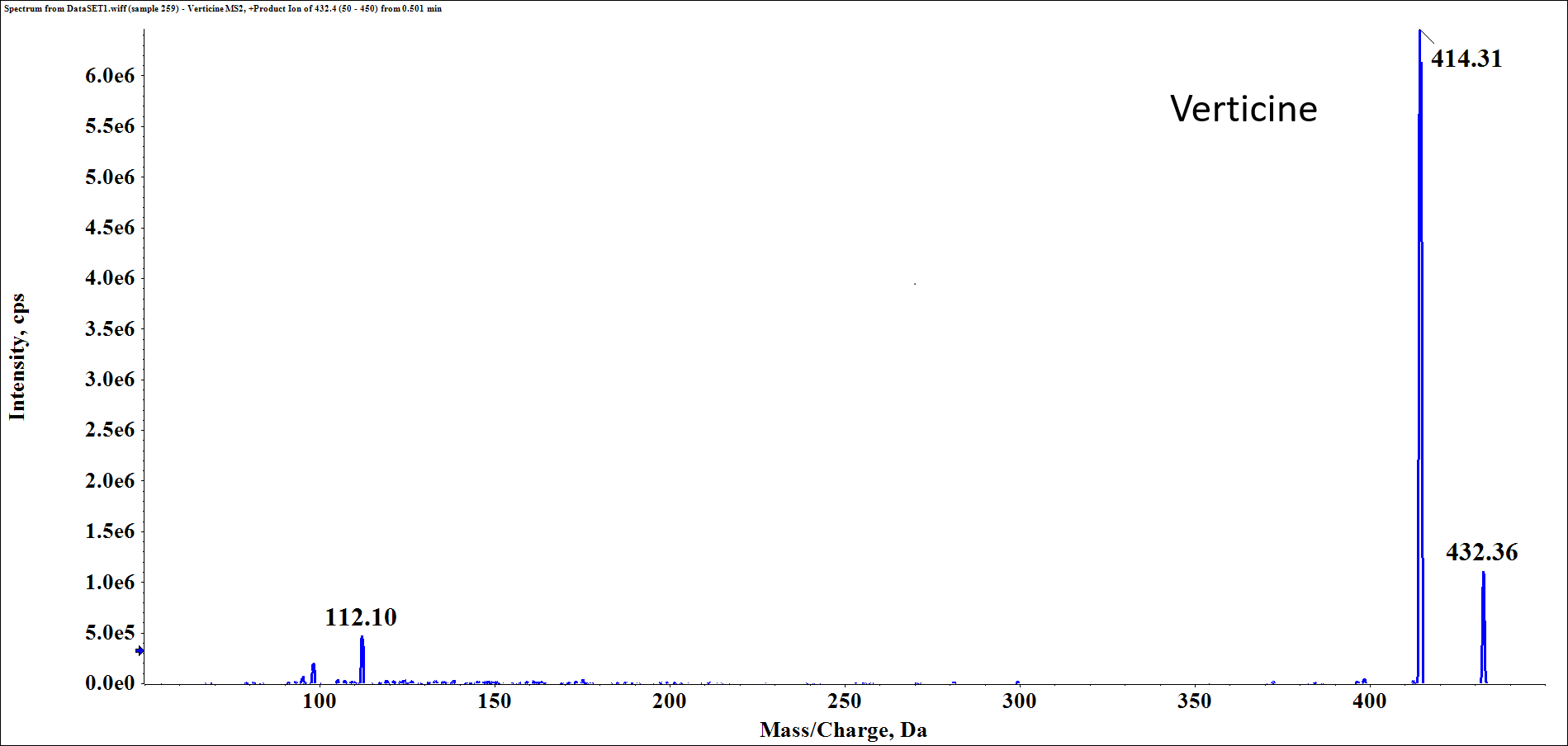


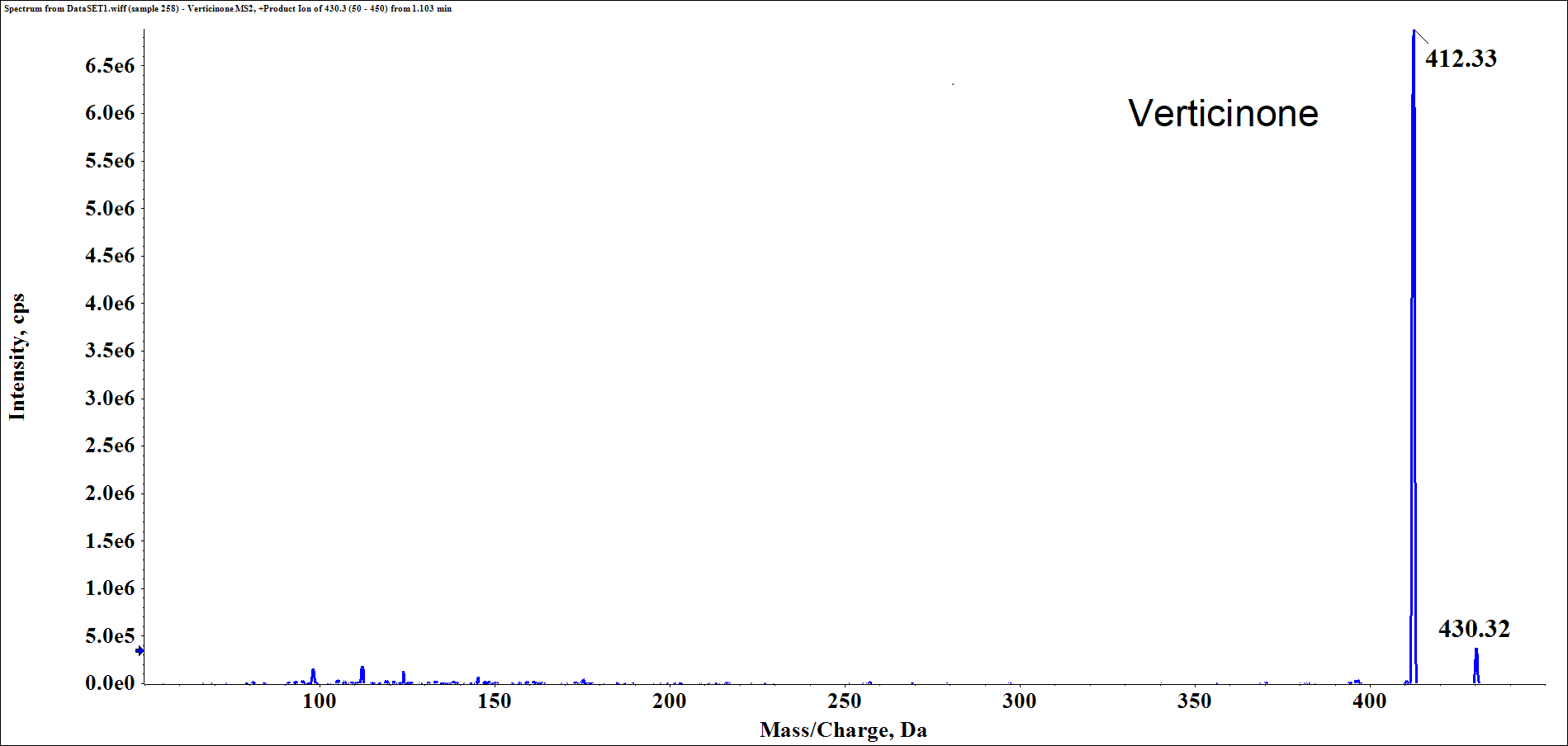


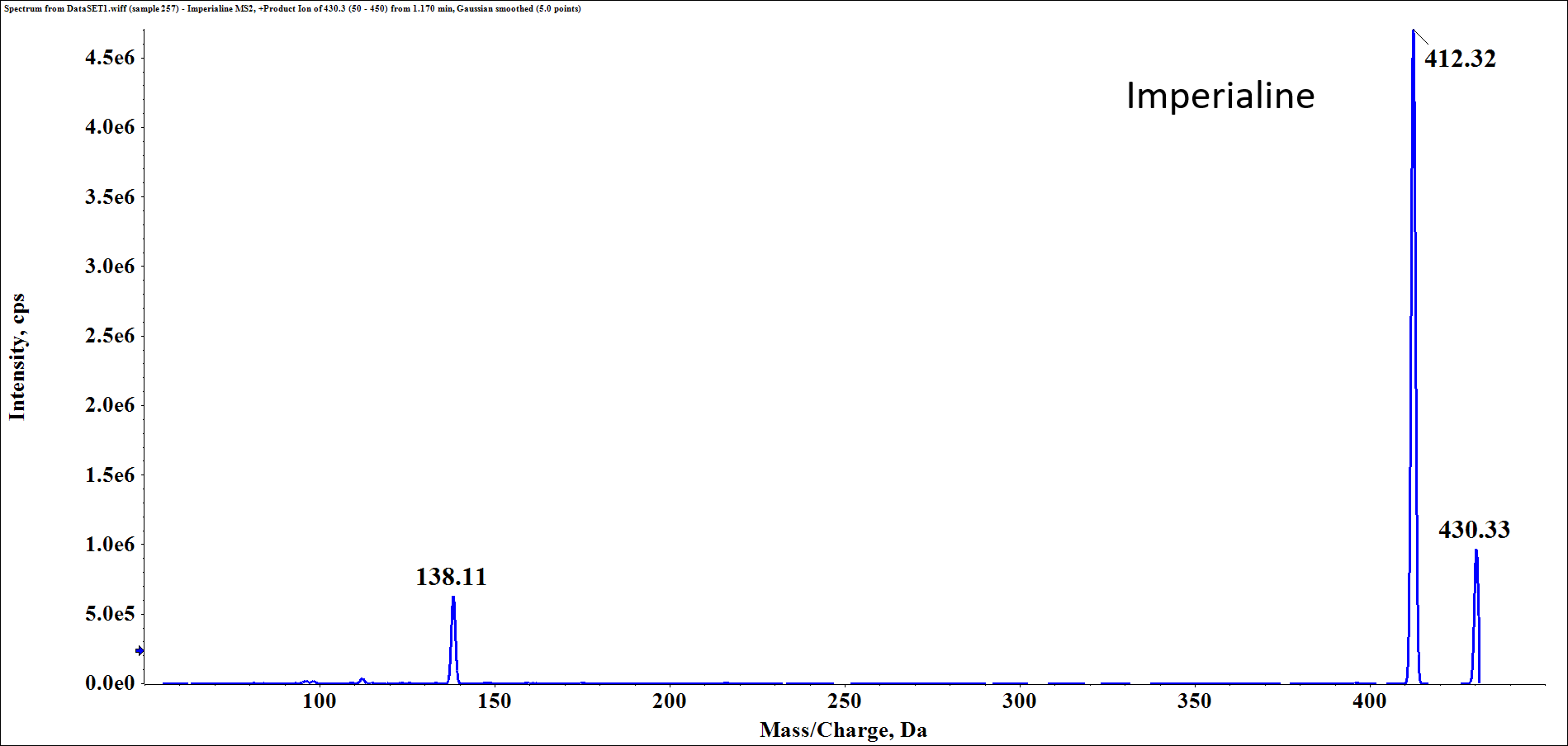
**Figure. S1** Mass spectrums of nine standard alkaloids.
